# Surface reconstruction of InAs (001) depending on the pressure and temperature examined by density functional thermodynamics

**DOI:** 10.1038/s41598-017-10881-2

**Published:** 2017-09-06

**Authors:** In Won Yeu, Jaehong Park, Gyuseung Han, Cheol Seong Hwang, Jung-Hae Choi

**Affiliations:** 1 Center for Electronic Materials, Korea Institute of Science and Technology, Seoul, 02792 Korea; 2 Department of Materials Science and Engineering and Inter-university Semiconductor Research Center, Seoul National University, Seoul, 08826 Korea

## Abstract

A detailed understanding of the atomic configuration of the compound semiconductor surface, especially after reconstruction, is very important for the device fabrication and performance. While there have been numerous experimental studies using the scanning probe techniques, further theoretical studies on surface reconstruction are necessary to promote the clear understanding of the origins and development of such subtle surface structures. In this work, therefore, a pressure-temperature surface reconstruction diagram was constructed for the model case of the InAs (001) surface considering both the vibrational entropy and configurational entropy based on the density functional theory. Notably, the equilibrium fraction of various reconstructions was determined as a function of the pressure and temperature, not as a function of the chemical potential, which largely facilitated the direct comparison with the experiments. By taking into account the entropy effects, the coexistence of the multiple reconstructions and the fractional change of each reconstruction by the thermodynamic condition were predicted and were in agreement with the previous experimental observations. This work provides the community with a useful framework for such type of theoretical studies.

## Introduction

The metal oxide semiconductor field effect transistor (MOSFET) is a key component in semiconductor devices that have been scaled down according to the well-known Moore’s law for the past half a century^1^. As the simple linear scaling of the physical gate length (L) of MOSFET has approached its physical limit, there were recently two major modifications of the material and device structure: the adoption of the high-k gate dielectric/metal gate in 2007^2^ and the three-dimensional channel structure, such as FinFET, in 2014^3^. Even with these innovations, L will keep decreasing, and at the L of 5 nm node, the presently prevailing Si channel will have to be replaced with a higher mobility channel material, such as InGaAs and Ge, for the n- and p-type MOSFETs, respectively. Nonetheless, these channel materials will not be used in a bulk single crystalline configuration but with a local heterogeneous epitaxial form on a Si substrate. Molecular beam epitaxy (MBE) or atomic layer epitaxy (ALE) must be the appropriate process for growing such layers, and numerous researches have been performed on this subject^4–6^.

Apart from the Si (or even Ge) single crystal surface, the compound semiconductors have inherently complicated surface structures, especially in terms of surface reconstruction. Surface reconstruction occurs to minimize the surface energy (γ) of a specific crystallographic plane by altering some of its bonding configurations, which will have a crucial influence on the dielectric film growth on top, and thus, on the device performance^7–10^. The film growth itself by either MBE or ALE can be influenced by the surface reconstruction, or conversely, the surface reconstruction can be affected by the film growth conditions^11–14^. Therefore, understanding these reconstruction behaviors under different environments is very important not only for the device fabrication and characterization but also for the theoretical understanding of the surface properties.

In this work, InAs was used as the prototypical compound semiconductor for the n-type MOSFET. Among the low index surfaces of InAs, there has been much interest on the (001) surface because a variety of surface reconstructions on (001) provide diverse controllability and because it is desirable to be grown on the commercial Si (001) wafer^4–6^. Many experimental studies confirmed that the dominant reconstruction of InAs (001) changes from c(4 × 4) to (2 × 4) and finally to (4 × 2), or vice versa, depending on the thermodynamic conditions^13–15^.

The density functional theory (DFT) calculations also examined the reconstructions of the InAs (001) surface^11, 16–19^. One of the inherent limitations of the conventional DFT calculations, however, is that they cannot deal with all the possible reconstructions using the limited-size supercell. For example, the DFT calculations failed to confirm any stable (4 × 2) reconstruction^11^ despite the experimental observation of the InAs (001) (4 × 2) reconstruction in the In-rich condition^13, 14, 20–22^. One of the plausible reasons for this is that certain unknown (4 × 2) reconstructions may be stable. In fact, several new InAs (001) (4 × 2) reconstructions were suggested, such as β3′(4 × 2)^20^, ζ(4 × 2)^17, 18^, and ζa(4 × 2)^19, 21, 22^, whose feasibilities are still being debated on. Another limitation of the conventional DFT calculations is that they cannot predict the coexistence of multiple reconstructions, although the coexistence of some reconstructions on InAs (001) was experimentally identified, such as the mixture of c(4 × 4) with (2 × 4)^15^, that of β2(2 × 4) with α2(2 × 4)^11–13^, and that of (2 × 4) with (4 × 2)^14^. The shortcoming of not being able to predict the coexistence of multiple reconstructions was overcome by considering the configurational entropy^23, 24^. These earlier theoretical works, however, showed the equilibrium fraction of each reconstruction as a function of the temperature (T) at a fixed chemical potential (μ), or as a function of μ at a fixed T. These calculation results are not highly comparable with those of the experimental procedure because μ can hardly be controlled directly but must be dealt with by changing to the pressure (P) and T. Therefore, predicting the surface reconstruction as a function of P and T is a much more preferred approach.

In this study, the equilibrium fraction of various reconstructions for a given P and T condition was predicted by taking into account both the vibrational entropy and configurational entropy using density functional thermodynamic calculations. The InAs (001) surface was studied as a model case. All the calculations were performed according to the following conceptual flow: the surface energy at 0K was calculated as a function of the chemical potential of As; then, the surface energy at 0K was converted to the function of P and T under the assumption of the thermodynamic equilibrium between the surface atom and the surrounding reservoir; then, the surface energy at non-0K was obtained as a function of P and T by considering the vibrational entropy; finally, the coexistence of the reconstructions was estimated by taking the configurational entropy into account. Unlike the previous calculations on the oxides with oxygen gas as the surrounding reservoir^25, 26^, the surrounding phase of InAs can be either an As solid or gaseous mixture composed of As_2_ and As_4_ molecules. Therefore, the procedure for calculating the (P-T) surface reconstruction diagram in InAs is more complicated. The calculated fraction of reconstructions and the (P-T) transition boundary were found to match those in the experimental reports on InAs (001)^13–15^. The methodology applied in this study can also be used for other surfaces and other materials to extend the understanding of surface reconstruction.

## Results and Discussions

The surfaces were represented by slabs consisting of eight or nine atomic layers with an at-least-10 Å vacuum layer. To eliminate the effect of the dipole moment along the vacuum, dipole correction^27^ was used. A preliminary calculation was carried out for a slab with both surfaces passivated by pseudo-hydrogen atoms (Z_H_ = 1.25), where only the hydrogen atoms were allowed to move and the total energy (${E}_{tot}^{{\rm{I}}}$) was obtained. From this calculation, the sum of the hydrogen-passivated surface energy (${\gamma }_{H}$), including the atomic energy of the pseudo-hydrogen and the bonding energy of the pseudo-hydrogen with In atoms on both ends (α), was obtained using the following equation:

1 $${\gamma }_{H}+\alpha =\frac{[{E}_{tot}^{{\rm{I}}}-{N}_{In}^{{\rm{I}}}{\mu }_{In(InAs)}-{N}_{As}^{{\rm{I}}}{\mu }_{As(InAs)}]}{2A}$$

where ${\mu }_{In(InAs)}$, ${\mu }_{As(InAs)}$, ${N}_{In}^{{\rm{I}}}$, ${N}_{As}^{{\rm{I}}}$, and $A$ are the chemical potentials of In and As in InAs, the numbers of In and As atoms, and the cross-sectional area of the slab, respectively.

Through the thermodynamic equilibrium, ${\mu }_{In(InAs)}$ and ${\mu }_{As(InAs)}$ vary with the following constraints:

2 $${\mu }_{In(InAs)}+{\mu }_{As(InAs)}={\mu }_{InAs(bulk)}$$

3 $${\mu }_{In(InAs)}\le {\mu }_{In(bulk)}\,and\,{\mu }_{As(InAs)}\le {\mu }_{As(bulk)}$$

By combining the equations (2) and (3):

4 $${\mu }_{InAs(bulk)}-{\mu }_{In(bulk)}\le {\mu }_{As(InAs)}\le {\mu }_{As(bulk)}$$

where, ${\mu }_{InAs(bulk)}$, ${\mu }_{In(bulk)}$, and ${\mu }_{As(bulk)}$ are the chemical potentials in the bulk solid states. Note that, ${\mu }_{As(InAs)}$ is constrained by the upper limit of ${\mu }_{As(bulk)}$ and the lower limit of ${\mu }_{InAs(bulk)}-{\mu }_{In(bulk)}$, respectively, and that the upper and lower limits are not constant, but decrease as T increases. The detailed calculations are explained in the on-line supplementary information (SI).

At 0K, the equation (4) becomes:

5 $${\mu }_{InAs(bul{k}^{0K})}-{\mu }_{In(bul{k}^{0K})}\le {\mu }_{As(InAs)}\le {\mu }_{As(bul{k}^{0K})}$$

where, ${\mu }_{InAs(bul{k}^{0K})}$, ${\mu }_{In(bul{k}^{0K})}$, and ${\mu }_{As(bul{k}^{0K})}$ are the energy of the bulk solid states at 0K. The μ for the bulk solid states at 0K was calculated using the tetragonal phase of In, the rhombohedral phase of As, and the zinc blende phase of InAs, respectively. Then, the equation (1) can be described as a function of ${\mu }_{As(InAs)}$ through the substitution of equation (2):

6 $${\gamma }_{H}+\alpha =\frac{[{E}_{tot}^{{\rm{I}}}-{N}_{In}^{{\rm{I}}}{\mu }_{InAs(bul{k}^{0K})}-({N}_{As}^{{\rm{I}}}-{N}_{In}^{{\rm{I}}}){\mu }_{As(InAs)}]}{2A}$$

For the calculation of the reconstruction, the passivating hydrogen atoms on the top surface were eliminated, and the atoms at the top five layers were relaxed, while the other bottom layers and passivating hydrogen atoms at the bottom surface were fixed until the remaining forces were less than 0.02 eV/Å. Then, $\gamma$ at 0K, ${\gamma }^{0{\rm{K}}}$ for each reconstruction was calculated as a function of ${\mu }_{As(InAs)}$ using the following equation:

7 $${\gamma }^{0{\rm{K}}}=\frac{[{E}_{tot}^{{\rm{II}}}-{N}_{In}^{{\rm{II}}}{\mu }_{InAs(bul{k}^{0K})}-({N}_{As}^{{\rm{II}}}-{N}_{In}^{{\rm{II}}}){\mu }_{As(InAs)}]}{A}-({\gamma }_{H}+\alpha )$$

where, ${E}_{tot}^{{\rm{II}}}$ is the total energy of this slab at 0K. It should be pointed out that ${\gamma }^{0{\rm{K}}}$ is the function only of ${\mu }_{As(InAs)}$ with the slope of $({N}_{In}^{{\rm{I}}{\rm{I}}}-{N}_{As}^{{\rm{I}}{\rm{I}}})$, and that the other values in equation (7) are all fixed for the given slab model.

**Figure 1:**
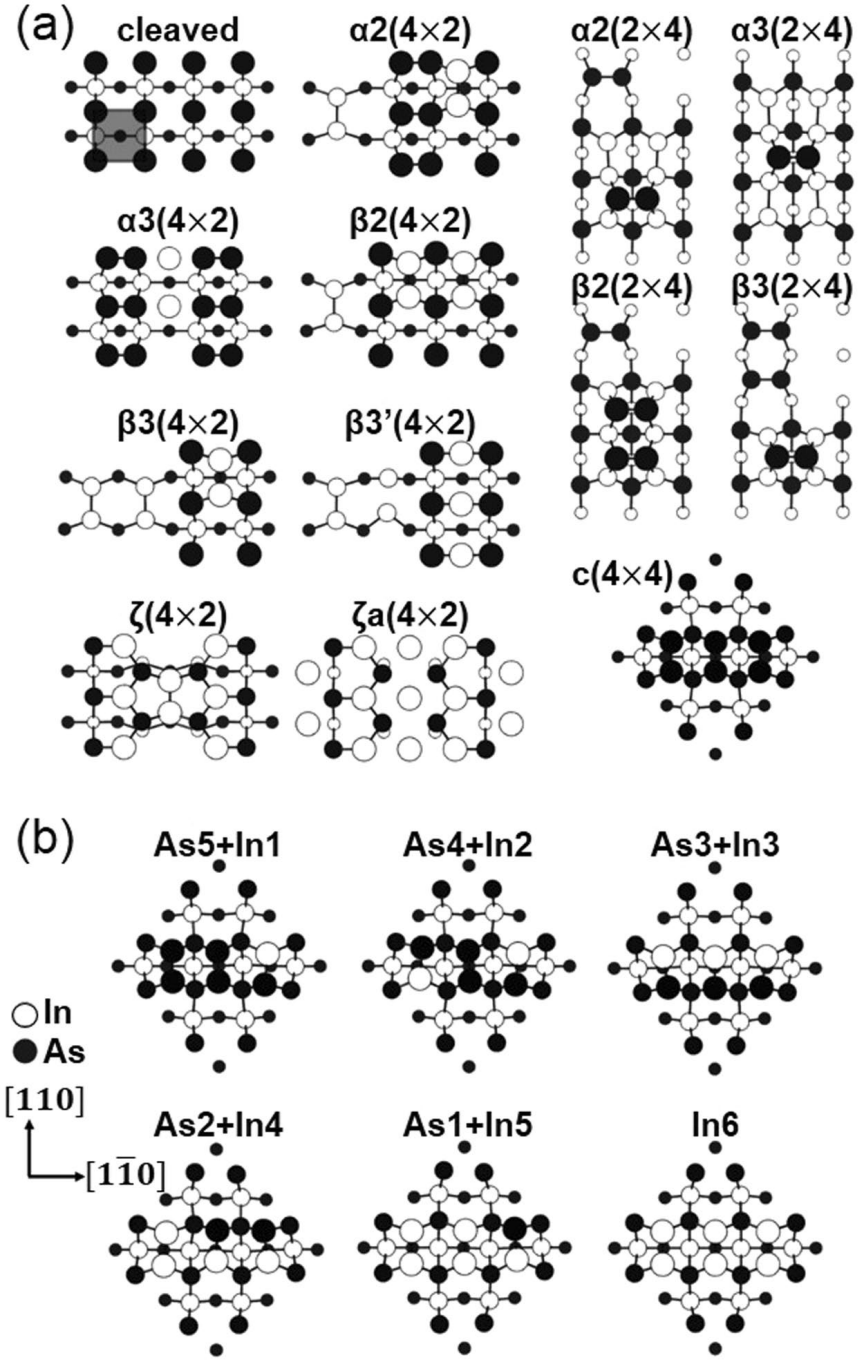
(**a**) Top view of the reconstructions of the InAs (001) surface compared with the cleaved surface (**b**) Top view of the c(4 × 4) heterodimers of various surface compositions. For example, ‘As5+In1’ means that the topmost layer is composed of five As atoms and one In atom. These configurations show the lowest surface energy for a given surface composition. All the surface areas correspond to their surface unit cell size except the cleaved surface whose surface unit cell size is indicated by a shaded square. The largest circles indicate the topmost layer.

Figure 1(a) shows the top view of the calculated reconstructions of InAs (001). For reference, the as-cleaved As-terminated surface structure was also included. The more the reconstructions included in the calculations, the more accurate the prediction of the fraction of each reconstruction. Therefore, all the previously suggested (4 × 2) reconstructions, such as β3′(4 × 2)^20^, ζ(4 × 2)^17, 18^, and ζa(4 × 2)^19, 21, 22^, were examined. In addition, for the c(4 × 4) reconstructions, not only the homodimers consisting of the six surface As atoms but also the heterodimer configurations were considered, as shown in Fig. 1(b). All the possible heterodimer configurations were constructed by replacing one to six As atoms at the top layer with In atoms, as summarized in Table 1. For example, ‘As5+In1’ corresponds to the topmost dimer layer composed of five As atoms and one In atom. The reconstructions in Fig. 1(b) show the structures with the lowest surface energy for a given surface composition.

**Table 1:** Two-dimensional symmetry group (2SG), the number of symmetry operations (σ), and degeneracy factor (g) for the various reconstructions of InAs (001). For the c(4 × 4) heterodimers, the configuration showing the lowest surface energy for a given surface composition is in bold.

Reconstruction	2SG	σ	$\frac{{\boldsymbol{g}}}{{{\boldsymbol{\sigma }}}_{(1\times 1)}}$	Reconstruction	2SG	σ	$\frac{{\boldsymbol{g}}}{{{\boldsymbol{\sigma }}}_{(1\times 1)}}$
α2(4 × 2)	p1	1	8	ζa(4 × 2)	pmm	4	2
αα3(4 × 2)	pmm	4	2	α2(2 × 4)	p1	1	8
β2(4 × 2)	pm	2	4	α3(2 × 4)	pmm	4	2
β3(4 × 2)	pm	2	4	β2(2 × 4)	pm	2	4
β3′(4 × 2)	p1	1	8	β3(2 × 4)	pm	2	4
ζ(4 × 2)	pmm	4	2	c(4 × 4)	cmm	8	2
c(4 × 4) As6	cmm	8	2	c(4 × 4) As3+In3(d)	p1	2	8
**c(4 × 4) As5+In1(a)**	p1	2	8	c(4 × 4) As3+In3(e)	p1	2	8
c(4 × 4) As5+In1(b)	cm	4	4	c(4 × 4) As3+In3(f)	p1	2	8
**c(4 × 4) As4+In2(a)**	p2	4	4	c(4 × 4) As2+In4(a)	p2	4	4
c(4 × 4) As4+In2(b)	p1	2	8	c(4 × 4) As2+In4(b)	p1	2	8
c(4 × 4) As4+In2(c)	cm	4	4	c(4 × 4) As2+In4(c)	cm	4	4
c(4 × 4) As4+In2(d)	p1	2	8	**c(4 × 4) As2+In4(d)**	p1	2	8
c(4 × 4) As4+In2(e)	cm	4	4	c(4 × 4) As2+In4(e)	cm	4	4
c(4 × 4) As4+In2(f)	cmm	8	2	c(4 × 4) As2+In4(f)	cmm	8	2
**c(4 × 4) As3+In3(a)**	cm	4	4	**c(4 × 4) As1+In5(a)**	p1	2	8
c(4 × 4) As3+In3(b)	p1	2	8	c(4 × 4) As1+In5(b)	cm	4	4
c(4 × 4) As3+In3(c)	cm	4	4	c(4 × 4) In6	cmm	8	2

**Figure 2:**
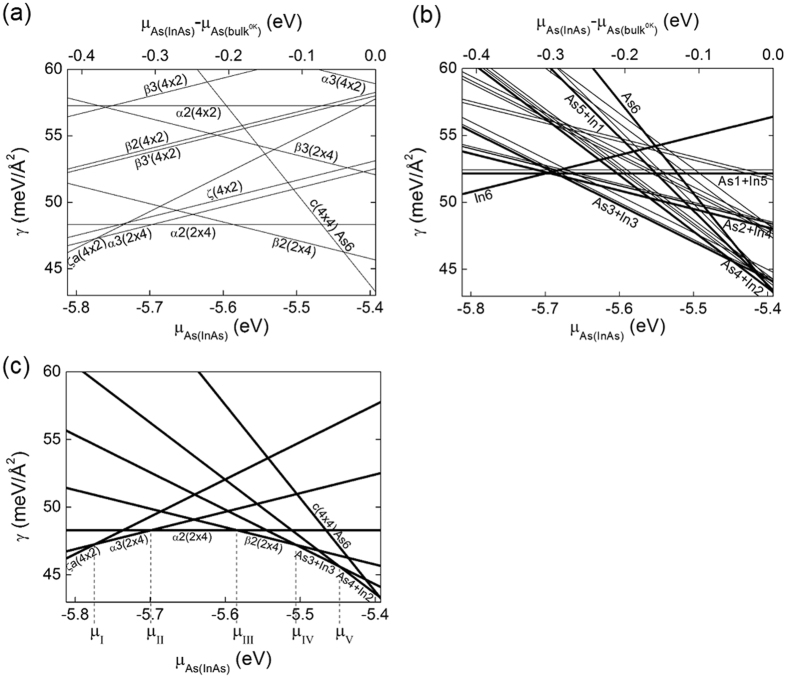
(**a**) Calculated surface energy at 0K for the InAs (001) reconstructions shown in Fig. 1(a) and (b) c(4 × 4) composed of heterodimers as well as homodimers (=As6). (**c**) Low values in (**a**) and (**b**).

Figure 2(a) shows the calculated ${\gamma }^{0{\rm{K}}}$ of the InAs (001) reconstructions shown in Fig. 1(a), as a function of both $[{\mu }_{As(InAs)}-{\mu }_{As(bul{k}^{0K})}]$ (top-axis) and that of ${\mu }_{As(InAs)}$ (bottom-axis), respectively. The top-axis representation is conventional as in the previous reports^11, 16–19^, while the bottom-axis representation was added for ease of conversion from $\mu$ to the corresponding (P-T) in the latter part of this study. Note that both ranges satisfy equation (5) and the slope in γ as a function of ${\mu }_{As(InAs)}$ is proportional to the excess number of In atoms compared to the As atoms in the slab, as represented in equation (7). Therefore, the fact that the slope of the most stable reconstruction decreases as ${\mu }_{As(InAs)}$ increases shows a consensus with the general conjecture that the reconstruction with more excess As atoms is more stable as ${\mu }_{As(InAs)}$ increases. In the middle of the ${\mu }_{As(InAs)}$ regime, the (2 × 4) reconstructions, such as α3(2 × 4), α2(2 × 4), and β2(2 × 4), are the most stable, and their positive, zero, and negative slopes correspond to their deficient, identical, and excess number of As atoms compared to the In atoms, respectively. In the In-rich condition (i.e., the low ${\mu }_{As(InAs)}$ regime), ζa(4 × 2) with the steepest slope was identified as the reconstruction with the lowest surface energy. On the contrary, in the As-rich condition (i.e., the high ${\mu }_{As(InAs)}$ regime), c(4 × 4) with a negative slope is the most stable.

Figure 2(b) shows the calculated ${\gamma }^{0{\rm{K}}}$ of the c(4 × 4) As6, which is reproduced from Fig. 2(a) and indicated as As6, and the other c(4 × 4) reconstructions with As-In heterodimers as a function of both $[{\mu }_{As(InAs)}-{\mu }_{As(bul{k}^{0K})}]$ and ${\mu }_{As(InAs)}$, respectively. The several lines for a certain slope denote the multiple configurations for a certain surface composition summarized in Table 1 and the lowest and thick line for a given slope correspond to the configuration in Fig. 1(b). In general, as ${\mu }_{As(InAs)}$ increases, the c(4 × 4) dimers with more As atoms become more stable, as can be expected, from ‘In6’ to ‘As3+In3’, then to ‘As4 + In2’. Only the structure of ‘In6’ shows a positive slope corresponding to its excess In atoms in the slab, while that of ‘As1+In5’ shows zero slope, indicating its overall stoichiometry. All the others’ negative slopes represent the excess As atoms in the slab. Note that in most of the ${\mu }_{As(InAs)}$ regime, the c(4 × 4) heterodimers remarkably reduced ${\gamma }^{0{\rm{K}}}$ values compared to the c(4 × 4) homodimer.

In Fig. 2(c), only the reconstructions with low ${\gamma }^{0{\rm{K}}}$ values in Fig. 2(a) and (b) are shown. The critical ${\mu }_{As(InAs)}$ values at which the ${\gamma }^{0{\rm{K}}}$ values of the two stable reconstructions are identical were set as μ_I_, μ_II_, μ_III_, μ_IV_, and μ_V_ respectively. For example, at μ_I_, the ${\gamma }^{0{\rm{K}}}$ of the ζa(4 × 2) is the same as that of the α3(2 × 4). Overall, the c(4 × 4) heterodimers with low ${\gamma }^{0{\rm{K}}}$ values expand the stable regime of c(4 × 4) compared to the c(4 × 4) homodimers.

Although the calculation results shown in Fig. 2 are extensive, their usefulness compared with the results of the experiments is limited because ${\gamma }^{0{\rm{K}}}$ was given as a function of ${\mu }_{As(InAs)}$, not as a function of P and T. To draw the (P-T) surface reconstruction diagram of the InAs (001) surface, ${\mu }_{As(InAs)}$ should be calculated as a function of P and T, which is certainly achievable from the assumption of the thermodynamic equilibrium between the surface atom and the surrounding reservoir. The bulk states of As can be regarded as the surrounding reservoir and present either in a solid state with the rhombohedral crystalline structure or in two forms of gaseous states: As_2_ and As_4_. Liquid phase As was ruled out because the triple point of As appears at 35.8 atm and 1090K while the growth conditions of the thin film in the MBE chamber are a high vacuum and a temperature of around 750K^11–13, 28^. On the other hand, the pressure of In gas has been known to be ~1 order lower than that of As gas in the MBE chamber^29^ and hence can be ignored.

**Figure 3:**
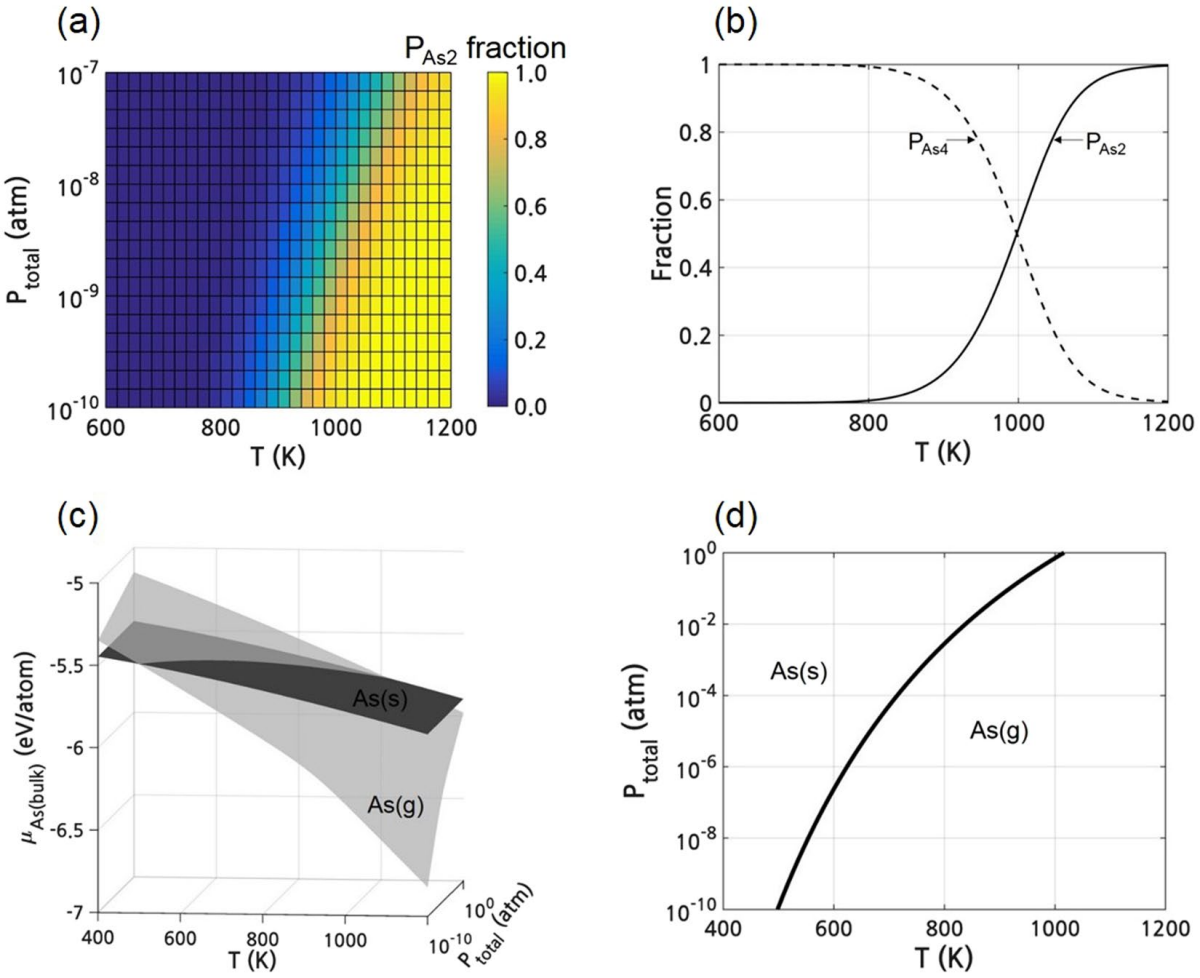
(**a**) Equilibrium fraction of P_As2_ as a function of T and P_total_(=P_As2_ + P_As4_). (**b**) Fractions of P_As2_ and P_As4_ as a function of T when the P_total_ is fixed at 4 × 10^−9^ atm. (**c**) Chemical potentials of arsenic solid (dark) and gas (light). (**d**) (P-T) phase diagram of pure As phase.

The relative stability of the surrounding reservoir was determined by calculating the Gibbs free energy of each phase. The Gibbs free energy of the gas phase was calculated considering the partial pressure of each gas under equilibrium. In the gas phase, the calculated equilibrium fraction of As_2_ increases as T increases or as P_total_ decreases, as shown in Fig. 3(a). Figure 3(b) shows the fractions of P_As2_ and P_As4_ as a function of T when P_total_ is fixed at 4 × 10^−9^ atm, which is comparable to the usual vacuum level for the MBE chamber^13, 14, 28^. In Fig. 3(c), both ${\mu }_{As(bulk)}$ in the solid phase and that in the gas phase were drawn as a function of P_total_ and T. For the solid phase, ${\mu }_{As(bulk)}$ decreases as T increases. On the contrary, it does not depend on P due to the assumption of the constant volume, which is a reasonable assumption because the volume change in solid is negligible compared to that in gas upon the variation of P. The phase boundary between the solid and gas phases in the (P-T) phase diagram of bulk As in Fig. 3(d) was obtained from the iso-chemical potential line between the solid and gas phases shown in Fig. 3(c).

**Figure 4:**
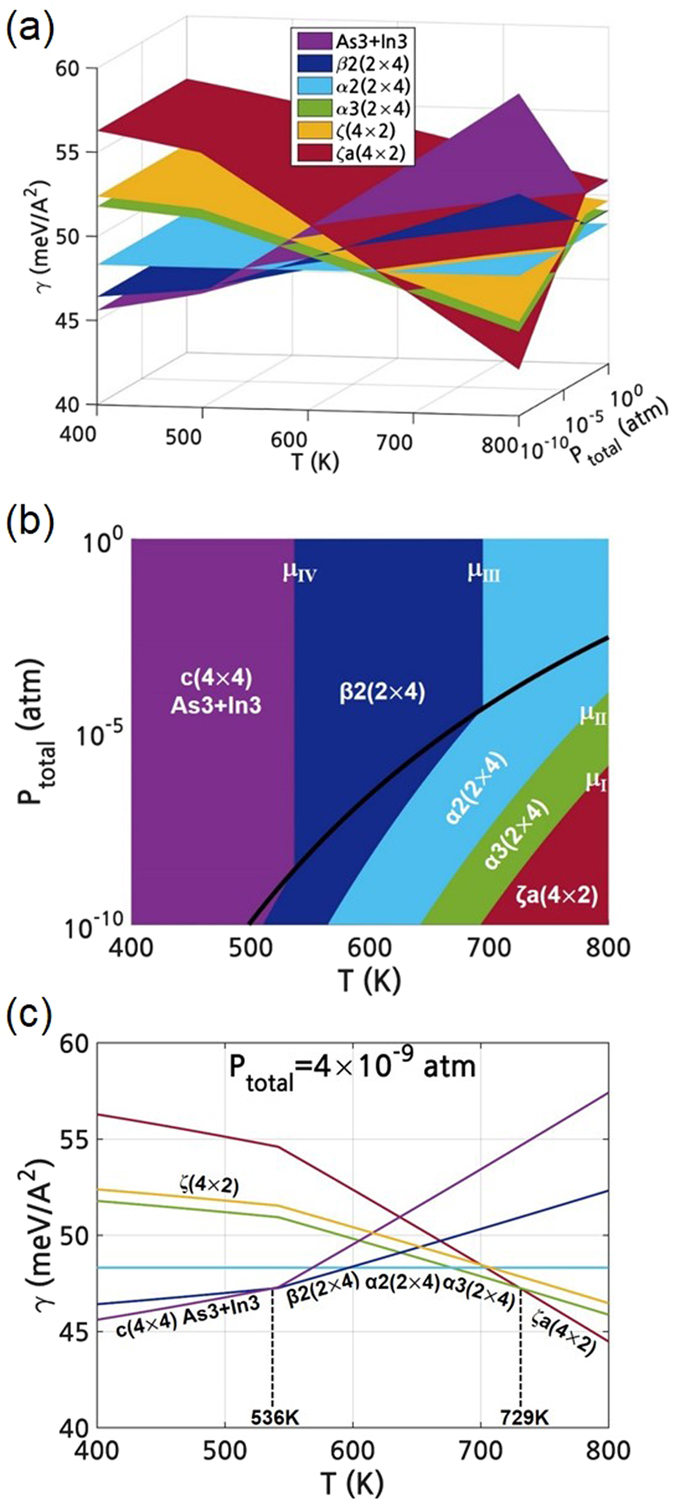
(**a**) Calculated surface energy of the InAs (001) reconstructions with low surface energy as a function of P and T. (**b**) (P-T) surface reconstruction diagram of the InAs (001) surface. (**c**) Calculated surface energy of the InAs (001) reconstructions with low surface energy as a function of T at fixed P_total_ of 4 × 10^−9^ atm. All figures were obtained without considering the surface phonons.

By combining the reconstruction with the lowest ${\gamma }^{0{\rm{K}}}$ as a function of ${\mu }_{As(InAs)}$ in Fig. 2(c) and ${\mu }_{As(bulk)}$ as a function of P and T in Fig. 3(c), Fig. 2(c) could be converted to the (P-T) region, as shown in Fig. 4(a). The conversion was done under the assumption that (i) the bulk As forms the surrounding reservoir of the InAs surface, (ii) the surrounding As is in thermodynamic equilibrium with the surface As in InAs (i.e., ${\mu }_{As(bulk)}={\mu }_{As(InAs)}$) and finally (iii) the $\gamma$ at non-0K, ${\gamma }^{{\rm{T}}}$ is almost the same as the ${\gamma }^{0{\rm{K}}}$ or critical ${\mu }_{As(InAs)}$ values of the ${\gamma }^{{\rm{T}}}$ is not significantly different from those of the ${\gamma }^{0{\rm{K}}}$. By doing so, the dependence of ${\gamma }^{0{\rm{K}}}$ on ${\mu }_{As(InAs)}$ is converted to the dependence on ${\mu }_{As(bulk)}$, and finally to that on (P-T) owing to the dependence of ${\mu }_{As(bulk)}$ on the (P-T) as shown in Fig. 3(c). Note that Fig. 4(a) is the direct conversion of Fig. 2(c).

Figure 4(b) is the surface reconstruction phase diagram of InAs (001) and shows the reconstruction with the lowest surface energy at a given (P-T), which is the bottom view of Fig. 4(a). Transition lines between different reconstructions correspond to the critical ${\mu }_{As(InAs)}$ values such as μ_I_~ μ_IV_ in Fig. 2(c) where the reconstruction having the lowest surface energy changes. As the stable state of bulk As is either solid or gas depending on P and T, as shown in Fig. 3(d), the (P-T) region in Fig. 4(b) was also divided into two regions according to the surrounding phase of either solid or gas. The bold curve in Fig. 4(b) is the phase boundary line between the bulk solid and gas phases of As, which are identical in the phase boundary curve indicated in Fig. 3(d). Therefore, some lines such as μ_III_ and μ_IV_ are partially not dependent on P where the solid As is the surrounding reservoir of the InAs surface due to the assumption of the constant volume of solid.

Figure 4(c) shows the transition of the surface reconstruction at P_total_ of 4 × 10^−9^ atm; c(4 × 4) ‘As3+In3’ → β2(2 × 4) → α2(2 × 4) → α3(2 × 4) → ζa(4 × 2) as T increases. These tendencies are consistent with the previous scanning tunneling microscopy (STM) observations showing the dependence of the InAs (001) reconstructions on the annealing temperature^11–15^. The transition temperature from c(4 × 4) to (2 × 4) and from (2 × 4) to (4 × 2) was calculated as 536K and 729K, respectively. These calculated transition temperatures are slightly lower but are in feasible agreement with the experimental values of 550K^15^ and 750K^13, 14^ in the UHV condition (~4 × 10^−9^ atm), respectively.

It is worth emphasizing that the low P and high T refer to the low ${\mu }_{As(bulk)}$($={\mu }_{As(InAs)}$). Therefore, such a change in the dominant reconstruction as a function of P and T is in direct accordance with the changes in the lowest ${\gamma }^{0{\rm{K}}}$ as a function of ${\mu }_{As(InAs)}$ in Fig. 2(c) (from right to left). Moreover, the conversion of the variation of ${\mu }_{As(InAs)}$ to the variation of P and T makes it practical to directly compare the calculation data with the experimental observations. The procedure explained so far was performed under the assumption that the ${\gamma }^{{\rm{T}}}$ is almost the same as the ${\gamma }^{0{\rm{K}}}$ or the variations of the surface energy caused by the surface vibrational entropy for the various reconstructions are the same. In InAs, however, the differences in the bonding geometries and stoichiometry of the various surface reconstructions as well as the discrepancy in the mass between In and As are non-trivial, which might induce the different T dependence of various surface reconstructions. Therefore, the surface vibrational entropy was also taken into account. In fact, in some semiconductors, the vibrational entropy of the surface plays a critical role in the energetics and properties^30, 31^.

For the calculation of the surface vibration, the atoms of the top three layers were treated as coupled harmonic oscillators and these atoms were displaced from their equilibrium positions. Under the assumption that the vibration of the atoms below these three topmost layers are the same as that of the atoms in the bulk state, the ${\gamma }^{{\rm{T}}}$ for each reconstruction was calculated using the following equation:

8 $${\gamma }^{{\rm{T}}}={\gamma }^{0{\rm{K}}}+\frac{[{F}_{vib,surf}^{{\rm{II}}}-{N}_{In,surf}^{{\rm{II}}}{F}_{vib,InAs(bulk)}-({N}_{As,surf}^{{\rm{II}}}-{N}_{In,surf}^{{\rm{II}}}){F}_{vib,As(bulk)}]}{A}$$

where ${F}_{vib,surf}^{{\rm{II}}}$ is the vibrational free energy of the three topmost layers, ${N}_{In,surf}^{{\rm{II}}}$ and ${N}_{As,surf}^{{\rm{II}}}$ are the numbers of In and As atoms on the three topmost layers, ${F}_{vib,InAs(bulk)}$ and ${F}_{vib,As(bulk)}$ are the vibrational free energy of the bulk InAs and bulk As, respectively. The detailed calculations of vibrational free energy and ${\gamma }^{{\rm{T}}}$ are explained in the computational methods and the on-line supplementary information (SI). The first term is the same as ${\gamma }^{0{\rm{K}}}$ in the equation (7) and the other terms were added to consider the difference between the effects of surface phonons and those of the bulk phonons.

**Figure 5:**
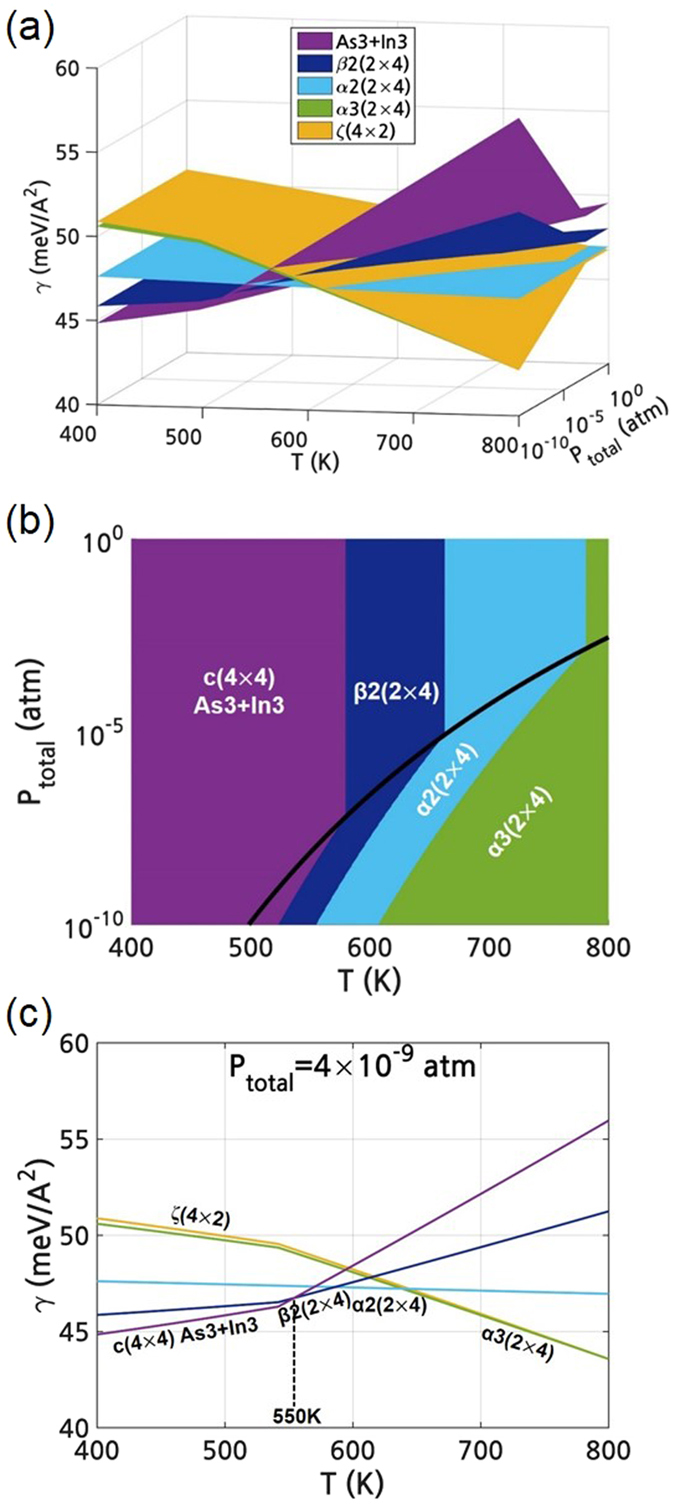
After considering the surface phonons, (**a**) calculated surface energy of the InAs (001) reconstructions with low surface energy as a function of P and T. (**b**) (P-T) surface reconstruction diagram of the InAs (001) surface. (**c**) Calculated surface energy of the InAs (001) reconstructions with low surface energy as a function of T at fixed P_total_ of 4 × 10^−9^ atm.

Figure 5(a) shows the ${\gamma }^{{\rm{T}}}$ as a function of P and T including the consideration of the surface vibration. The surface energy including the thermal effects, ${\gamma }^{{\rm{T}}}$, was converted to the (P-T) region as in Fig. 4. Note that the ζa(4 × 2) reconstruction was excluded in Fig. 5 due to its instability. The detailed data on the imaginary frequencies in the surface phonon DOS of the ζa(4 × 2) as well as the surface phonon DOSs for various reconstructions are shown in the on-line supplementary information (SI). Compared to Fig. 4(a), the surface energies of the all the reconstructions were entirely lowered and the transition lines between different reconstructions in Fig. 5(a) are changed by the thermal effects. The surface reconstruction diagram in Fig. 5(b) shows that the stable (P-T) region of the c(4 × 4) ‘As3+In3’ was especially enlarged compared to Fig. 4(b). Therefore, at P_total_ of 4 × 10^−9^ atm, the transition temperature between the c(4 × 4) and (2 × 4) was raised from 536K in Fig. 4(c) to 555K in Fig. 5(c). This calculated transition temperature is closer to the experimental values of 550K^15^. However, the experimentally found transition from (2 × 4) to (4 × 2) was not shown due to the exclusion of unstable ζa(4 × 2). By considering the surface vibrational entropy, on the other hand, the surface energy of the ζ(4 × 2) become closer to that of the α3(2 × 4) as can be compared in Figs. 4(c) and 5(c).

When some reconstructions show small energy difference, the reconstructions can coexist at the finite temperature due to the effects of the configurational entropy^23, 24^. The fraction of surface reconstruction j, ${f}_{j}$, can be determined by using the partition function of each reconstruction j, (${Z}_{j}$)^24^;

9 $${f}_{j}=\frac{{Z}_{j}}{Z},$$

where j $\in$ S (c(4 × 4), (2 × 4), (4 × 2), …; all calculated reconstructions)

10 $$Z=\sum _{j}{Z}_{j}=\sum _{j}{g}_{j}\exp (-\frac{{\gamma }_{j}A}{{k}_{B}T})$$

11 $${g}_{j}=\frac{{\sigma }_{(1\times 1)}}{{\sigma }_{(n\times m)}}{n}_{j}{m}_{j}$$

where, ${\gamma }_{j}$ is the surface energy of a certain reconstruction j, *A* is the surface area, ${k}_{B}$ is the Boltzmann constant, ${g}_{j}$ is the degeneracy factor related to the symmetry, ${n}_{j}$ and ${m}_{j}$ are the cell sizes of reconstruction j(n × m), and ${\sigma }_{(n\times m)}$ is the number of symmetry operations, as summarized in Table 1. It is noted that in this study, ${\gamma }_{j}$ was calculated as a function of P and T in Fig. 5; hence, ${f}_{j}$ can also be calculated as a function of P and T. The calculated ${f}_{j}$(P,T) enables the direct comparison with the experimental observations on the coexistence of multi-reconstructions as a function of P and T. It should be pointed out that this is a significant and more practical progress than the previous theoretical works^23, 24^ reporting ${f}_{j}\,\,$as a function of T at a fixed μ, or as a function of μ at a fixed T.

**Figure 6:**
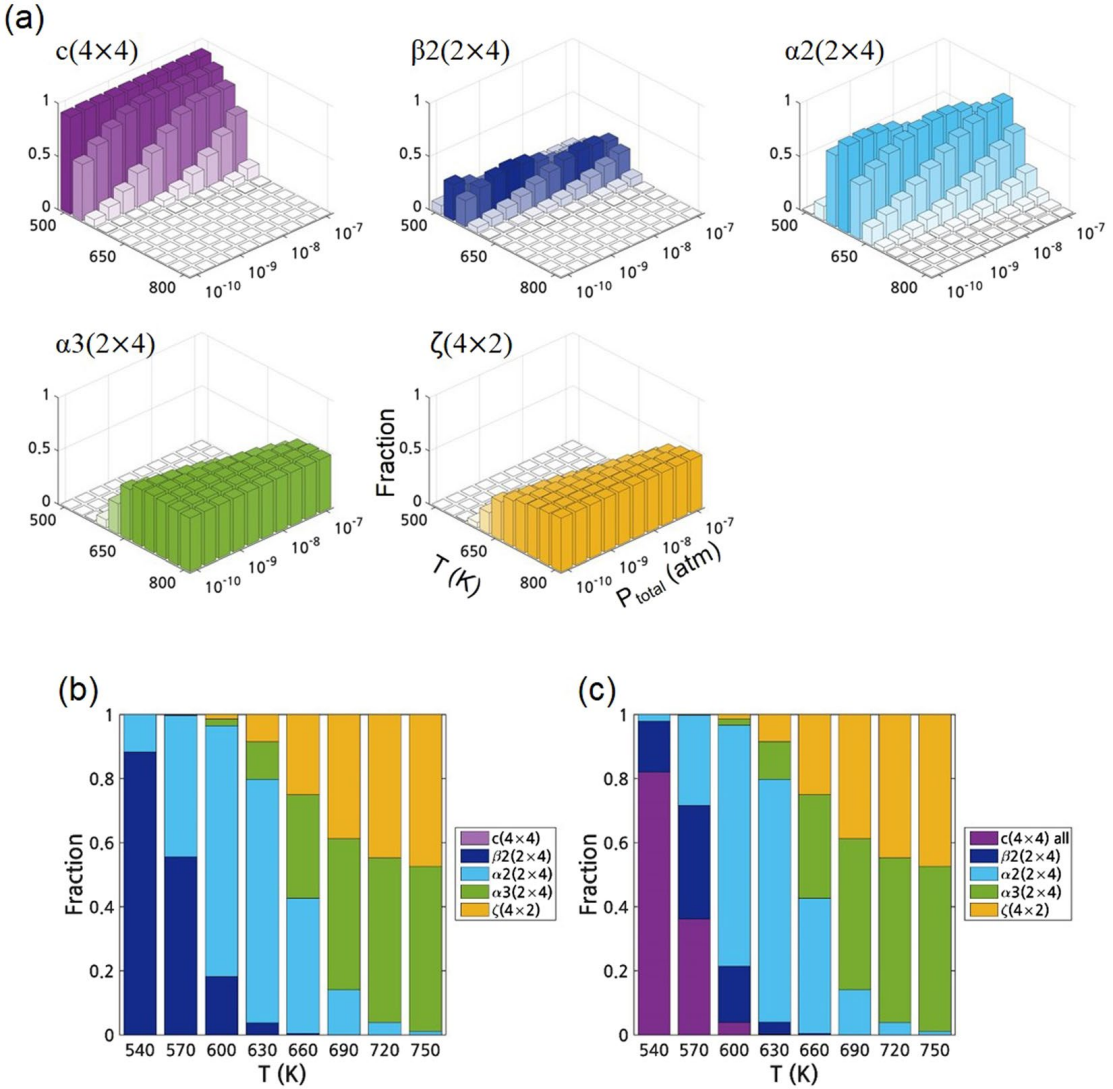
After considering both the surface phonons and configurational entropy, (**a**) the fraction of the dominant InAs (001) reconstructions. Fraction of the dominant InAs (001) reconstructions at a fixed P of 4 × 10^−9^ atm (**b**) without including the c(4 × 4) heterodimers, and (**c**) including all the c(4 × 4) heterodimers.

Figure 6(a) shows the equilibrium fraction of the dominant reconstructions on InAs (001) as a function of P and T. The ranges of P and T are from 10^−10^ to 10^−7^ atm and from 500 to 800K, respectively. The fraction of the c(4 × 4) includes that of pure As-dimers and all the heterodimers. The fraction of the other surfaces was not represented due to their negligible fraction. Figure 6(a) clearly shows the coexistence of the multiple reconstructions and the variation of the dominant reconstruction depending on P and T. As P decreases and T increases, the dominant reconstruction changed: c(4 × 4) → β2(2 × 4) → α2(2 × 4) → α3(2 × 4) → ζ(4 × 2) and the fraction of the ζ(4 × 2) is comparable to that of the α3(2 × 4).

Figure 6(b) and (c) show the fraction of reconstructions of InAs (001) as a function of T at a fixed P of 4 × 10^−9^ atm. For Fig. 6(b), the partition functions of only the reconstructions represented in Fig. 2(a) were included for the summation in equation (10), without considering the c(4 × 4) heterodimers. On the other hand, Fig. 6(c) was obtained by including all the reconstructions including all the c(4 × 4) heterodimers. These clearly demonstrate that the c(4 × 4) heterodimers boost the fraction of c(4 × 4) at the low T region and shows the transition of the dominant surface structures: c(4 × 4) → mixture of c(4 × 4) with (2 × 4) → (2 × 4) → mixture of (2 × 4) with (4 × 2) as T increases. The changes in the dominant reconstruction by T are in agreement with the previous experimental report that the transition from c(4 × 4) to (2 × 4)^15^ and from (2 × 4) to (4 × 2)^13, 14^ starts at around 550 and 750K at ~4 × 10^−9^ atm, respectively. When the configurational entropy are not considered, the direct transition from (2 × 4) to (4 × 2) does not appear in Fig. 5(c). However, the coexistence of the (2 × 4) and (4 × 2) is shown in Fig. 6(b) and (c) and the fractions of (2 × 4) and (4 × 2) become similar at 750K, which reflects the experimental transition T of 750K^13, 14^. In this study, by combining both the configurational entropy and vibrational entropy with the density functional thermodynamic calculations, the coexistence of the various reconstructions and the fractional change of each reconstruction in the coexistence region were successfully confirmed. Although the methodology of drawing the (P-T) surface reconstruction diagram was applied only to InAs (001) in this work, the same procedure can be readily applied to other surfaces and other materials to understand the surface reconstruction and to provide a guideline on the process window for the controlled thin film growth.

## Conclusion

For the InAs (001) surface, a pressure-temperature (P-T) surface reconstruction diagram was constructed by density functional calculations. The fundamental strategy is calculating the surface energy of all the probable surface reconstruction configurations as a function of the chemical potential of As, whose value is further estimated as a function of P and T based on the assumption of the thermodynamic equilibrium between the surface atom and the surrounding reservoir. The calculation also takes into account the multiple phases of As as the surrounding reservoir. In addition, the equilibrium fraction of various reconstructions was determined as a function of P and T by considering both the vibrational entropy and configurational entropy with the density functional thermodynamic calculations. At a total pressure of 4 × 10^−9^ atm, which is comparable to the typical molecular beam epitaxy (MBE) condition for InAs film growth, the c(4 × 4) reconstruction was the most favorable for T < ~550K, and (4 × 2) became dominant as T increased to over 750K. The prediction of the transition boundary and the fractional change during the transition using this diagram provides a direct method of comparing the calculation results with the experimental conditions, and showed good agreement with the previous experimental reports. This methodology can be applied to other surfaces and other materials to understand the surface reconstruction and to provide a guideline on the process window for controlled thin film growth.

## Computational Methods

### Surface energy at 0K

All the calculations were performed using Vienna *Ab-Initio* Simulation Package (VASP)^32–35^. The project augmented wave method^36, 37^ within the local density approximation (LDA) parameterized by Ceperley *et al*.^38, 39^ was used with a cutoff energy of 300 eV. The 4d, 5 s, and 5p orbitals for In, and the 4 s and 4p orbitals for As, were treated as the valence electrons. Slab geometries consisting of eight or nine atomic layers with an at-least-10 Å vacuum layer were relaxed until the forces were less than 0.02 eV/Å.

### Chemical potential of solid: ${{\boldsymbol{\mu }}}_{({\boldsymbol{solid}})}({\boldsymbol{P}},{\boldsymbol{T}})$

The μ of the bulk solids at non-0K was obtained from the Helmholtz free energy (F = E−TS_vib_) because the PV term in the Gibbs free energy (G = F + PV) is negligible due to the almost constant volume of solid, which is the general condition for the thin film growth of InAs. Harmonic oscillator approximation was used to calculate the vibration of crystal solid and the phonon density of states of solid was calculated using the finite displacement method implemented in PHONOPY^40, 41^.

### Chemical potential of gas: ${{\boldsymbol{\mu }}}_{({\boldsymbol{gas}})}({\boldsymbol{P}},{\boldsymbol{T}})$

The μ of the gas phase was calculated by considering the partition functions of the translational, rotational, vibrational, and electronic motions, respectively, as the following equation:

12 $${\mu }_{i(gas)}(P,T)=(-{k}_{B}T\,\mathrm{ln}\,{Q}_{i(gas)}^{tot}+PV)/N$$

13 $${Q}_{i(gas)}^{tot}=\frac{1}{N!}{({q}^{trans}{q}^{rot}{q}^{vib}{q}^{elec})}^{N}$$

14 $${\mu }_{i(gas)}(P,T)=-\frac{1}{N}[{k}_{B}T\,\mathrm{ln}(\frac{1}{N!}{({q}^{trans})}^{N})-PV]-{k}_{B}T\,\mathrm{ln}\,{q}^{rot}-{k}_{B}T\,\mathrm{ln}\,{q}^{vib}-{k}_{B}T\,\mathrm{ln}\,{q}^{elec}$$

where *P* is the partial pressure of i(gas), ${Q}_{i(gas)}^{tot}$ is the total partition function of i(gas) composed of N indistinguishable molecules, and ${q}^{trans},$ ${q}^{rot}$, ${q}^{vib}$, and ${q}^{elec}\,\,$are the translational, rotational, vibrational, and electronic partition functions of one i molecule. By substituting the partition function, the translation contribution to the chemical potential is given by:

15 $$-\frac{1}{N}[{k}_{B}T\,\mathrm{ln}(\frac{1}{N!}{({q}^{trans})}^{N})-PV]=-{k}_{B}T\,\mathrm{ln}[{(\frac{2\pi m}{{h}^{2}})}^{3/2}\frac{{({k}_{B}T)}^{5/2}}{{P}_{i(gas)}}]$$

where, *m* is the molecular mass, and *h* is the Planck constant, respectively. The rotation contribution is given by:

16 $$-{k}_{B}T\,\mathrm{ln}\,{q}^{rot}=-{k}_{B}T\,\mathrm{ln}(\frac{{\pi }^{\frac{1}{2}}}{\sigma }\sqrt{\frac{2{I}_{A}{k}_{B}T}{{\hbar}^{2}}}\sqrt{\frac{2{I}_{B}{k}_{B}T}{{\hbar}^{2}}}\sqrt{\frac{2{I}_{C}{k}_{B}T}{{\hbar}^{2}}})$$

where, ${I}_{A}$, ${I}_{B}$, and ${I}_{C}$ are the principal moments of inertia, *σ* is the symmetry number of the molecule, and *ħ* is $\frac{h}{2\pi }$. The vibration contribution is given by:

17 $$-{k}_{B}T\,\mathrm{ln}\,{q}^{vib}=\sum _{i=1}^{M}\frac{\hbar{w}_{i}}{2}+\sum _{i=1}^{M}{k}_{B}T\,\mathrm{ln}(1-{e}^{-\frac{\hbar{w}_{i}}{{k}_{B}T}})$$

where, *M* is the number of the vibrational normal modes (3 N–5 for linear molecules, and 3 N–6 for non-linear molecules, respectively) and ${w}_{i}$ is the corresponding frequency. Note that $\sum _{i=1}^{M}\frac{{\hbar}{w}_{i}}{2}$ term is the zero point energy of i(gas), ${E}_{i(gas)}^{ZPE}$. Finally, the electron contribution is given by:

18 $$-{k}_{B}T\,\mathrm{ln}\,{q}^{elec}={E}_{i(gas)}^{tot}-{k}_{B}T\,\mathrm{ln}\,{I}^{spin}$$

where, ${E}_{i(gas)}^{tot}$ is the total energy of the i(gas) and ${I}^{spin}$ is the electronic spin degeneracy of the ground state, respectively.

For ${\mu }_{i(gas)}(P,T)\,\,$in the equation (14), only the translation contribution expressed by the equation (15) is dependent on both P and T. The other contributions expressed by the equations (16), (17), and (18) are the functions only of T. By inserting the standard pressure, ${P}^{o}$(=1 atm), into the equation (15) to separate the dependency on P, the chemical potential of gas becomes:

19 $${\mu }_{i(gas)}(P,T)={E}_{i(gas)}^{tot}+{E}_{i(gas)}^{ZPE}+{\rm{\Delta }}{\mu }_{i(gas)}({P}^{o},T)+{k}_{B}T\,\mathrm{ln}(\frac{{P}_{i(gas)}}{{P}^{o}})$$

The first three terms in the right side of the equation (19) are defined as ${\mu }_{i(gas)}^{o}$, then

20 $${\mu }_{i(gas)}(P,T)={\mu }_{i(gas)}^{o}+{k}_{B}T\,\mathrm{ln}\,\frac{{P}_{i(gas)}}{{P}^{o}}$$

where, ${P}_{i(gas)}$ and ${\mu }_{i(gas)}^{o}$ are the partial pressure and the standard chemical potential of i(gas), respectively. ${\mu }_{i(gas)}^{o}$ is a function of T and was obtained from the DFT calculations on an isolated molecule in a 17.5 × 17.5 × 17.5 Å^3^ box. To the authors’ knowledge, ${\rm{\Delta }}{\mu }_{i(gas)}^{o}({P}^{o}=1\,atm,T)$ of As_2_ and As_4_ has not been reported experimentally. The calculated ${\rm{\Delta }}{\mu }_{i(gas)}^{o}({P}^{o},T)$ of O_2_, however, using the same scheme, was well matched with the thermodynamic data^42^.

Then, the two partial pressure, ${P}_{A{s}_{2}(gas)}$ and $\,{P}_{A{s}_{4}(gas)}$, can be calculated by solving:

21 $$\frac{1}{2}{\mu }_{A{s}_{4}}={\mu }_{A{s}_{2}}$$

22 $$\frac{1}{2}({\mu }_{A{s}_{4}(gas)}^{o}+{k}_{B}T\,\mathrm{ln}\,\frac{{P}_{A{s}_{4}(gas)}}{{P}^{o}})={\mu }_{A{s}_{2}(gas)}^{o}+{k}_{B}T\,\mathrm{ln}\,\frac{{P}_{A{s}_{2}(gas)}}{{P}^{o}}$$

23 $${P}_{A{s}_{2}}+{P}_{A{s}_{4}}={P}_{total}$$

at a given total pressure of P_total_ and T.

### Surface phonon

The surface phonons were calculated to predict the effects of the vibrational entropy by the frozen phonon approach. Starting from the relaxed surface reconstructions, the atoms of the three topmost layers were displaced from their equilibrium positions and the electronic energy was calculated until the energy difference between consecutive steps is less than 10^−6^ eV within the reconstructed supercell (~8.5 × 17 Å^2^). The resulting Hellmann-Feynman forces were used to determine the force constants by the harmonic approximation. The dynamical matrix was obtained at the 21 × 21 sampled k-points on the surface two-dimensional Brillouin zone. From the calculated frequencies at each k-points, ${w}_{i}(k)$, the surface vibration contribution to the energy was calculated by following equation:

24 $${F}_{vib}=\frac{1}{{N}_{k}}\sum _{k\in BZ}\sum _{i=1}^{M}\{\frac{\hbar{w}_{i}(k)}{2}+{k}_{B}T\,\mathrm{ln}(1-{e}^{-\frac{\hbar{w}_{i}(k)}{{k}_{B}T}})\}$$

where, ${N}_{k}$ is the number of k-points in the two-dimensional Brillouin zone.

## Electronic supplementary material

Supporting Information: SREP-17-14510
