# Supplementary material for: Surface reconstruction of InAs (001) depending on the pressure and temperature examined by density functional thermodynamics
Source: Sci Rep. 2017 Sep 6;7:10691. doi: 10.1038/s41598-017-10881-2 (PMC5587624; doi:10.1038/s41598-017-10881-2)
Supplement: Supplementary file 1 — Supporting Information: SREP-17-14510 [file 41598_2017_10881_MOESM1_ESM.pdf]

Supporting Information:

Surface reconstruction of InAs (001) depending on the pressure and temperature examined by density functional thermodynamics

In Won Yeu[a,b], Jaehong Park[a,b], Gyuseung Han[a,b], Cheol Seong Hwang[b], and Jung-Hae Choi[a,*]

[a]Center for Electronic Materials, Korea Institute of Science and Technology, Seoul 02792, Korea

[b]Department of Materials Science and Engineering and Inter-university Semiconductor Research Center, Seoul National University, Seoul 08826, Korea

[*]Corresponding author. Tel.: +82 2 958 5488; Fax: +82 2 958 6658.

E-mail address: choijh@kist.re.kr (J.-H. Choi).

1. Range of chemical potential of As in InAs

The chemical potential of As in InAs, $\mu_{As(InAs)}$ at arbitrary T is constrained by the upper limit of $\mu_{As(bulk)}$ and the lower limit of $\mu_{InAs(bulk)} - \mu_{In(bulk)}$, respectively.

$$\mu_{InAs(bulk)} - \mu_{In(bulk)} \leq \mu_{As(InAs)} \leq \mu_{As(bulk)} \quad (4)$$

where, $\mu_{InAs(bulk)}$, $\mu_{In(bulk)}$, and $\mu_{As(bulk)}$ are the chemical potentials in the bulk solid states. Because the PV term in the Gibbs free energy is negligible in the low P conditions, the chemical potentials in the bulk solid states are calculated using the following equations:

$$\mu_{(bulk)} = \frac{E_{tot}+F_{vib}}{N} \quad \text{(S. 1)}$$

$$\mathrm{F_{vib}} = \frac{1}{N_k}\sum_{k\in BZ}\sum_{i=1}^{M}\{\frac{\hbar w_i(k)}{2} + k_B T \ln\left(1 - e^{-\frac{\hbar w_i(k)}{k_B T}}\right)\} \quad (24)$$

where, E_{tot} is the total internal energy, N the number of atoms in the unit cell, and N_k is the number of k-points in the Brillouin zone.

The lower and upper limits of $\mu_{As(InAs)}$, shown in Fig. S1 (a) and (b) decrease as T increases. For Fig. 2, the lower and upper limits were arbitrarily chosen to those at 0K, and the surface energy at 0K was shown in this range for the ease of viewing. However, the wider range of $\mu_{As(InAs)}$ was considered to convert the $\mu_{As(InAs)}$ to the corresponding (P-T) in the later part.

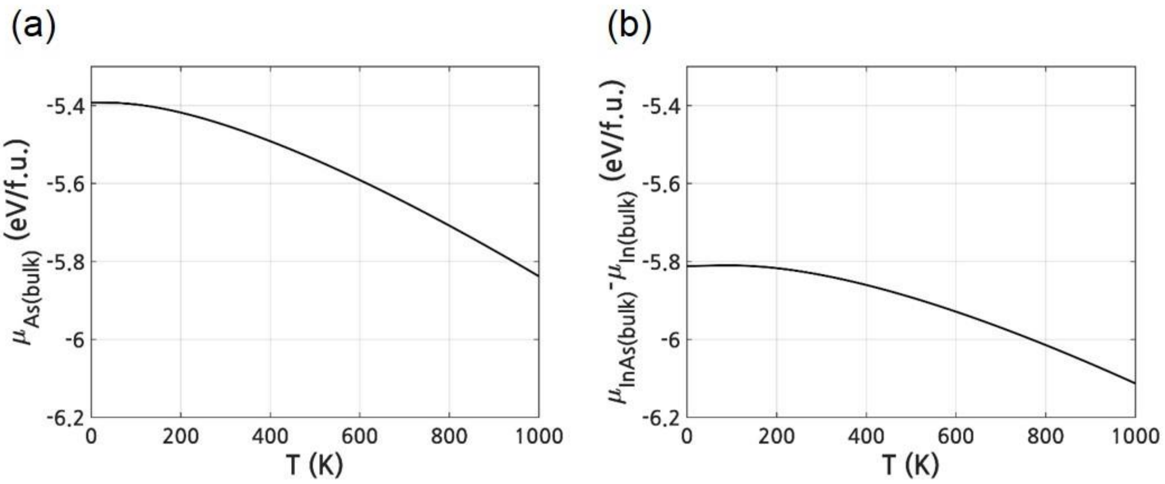

Figure S1. Calculated (a) lower limit and (b) upper limit of $\mu_{As(InAs)}$ as a function of T.

2. Surface vibrational properties

In order to verify the effects of the vibrational entropy on the surface, the surface phonons were calculated by frozen-phonon method. The phonon DOSs were plotted by smearing the frequencies using the Gaussian smearing method with σ=0.1. Figure S2 shows the calculated surface phonon DOSs of the various InAs (001) reconstructions. Only the reconstructions with low surface energy are shown. Note that the ζa(4×2) has some imaginary frequencies which indicates that the surface structure is not stable. The atomic structure of the ζa(4×2) is different from that of ζ(4×2) in that there are more In atoms on the topmost layer and the middle In dimer is broken, as shown in Fig. 1 (a).

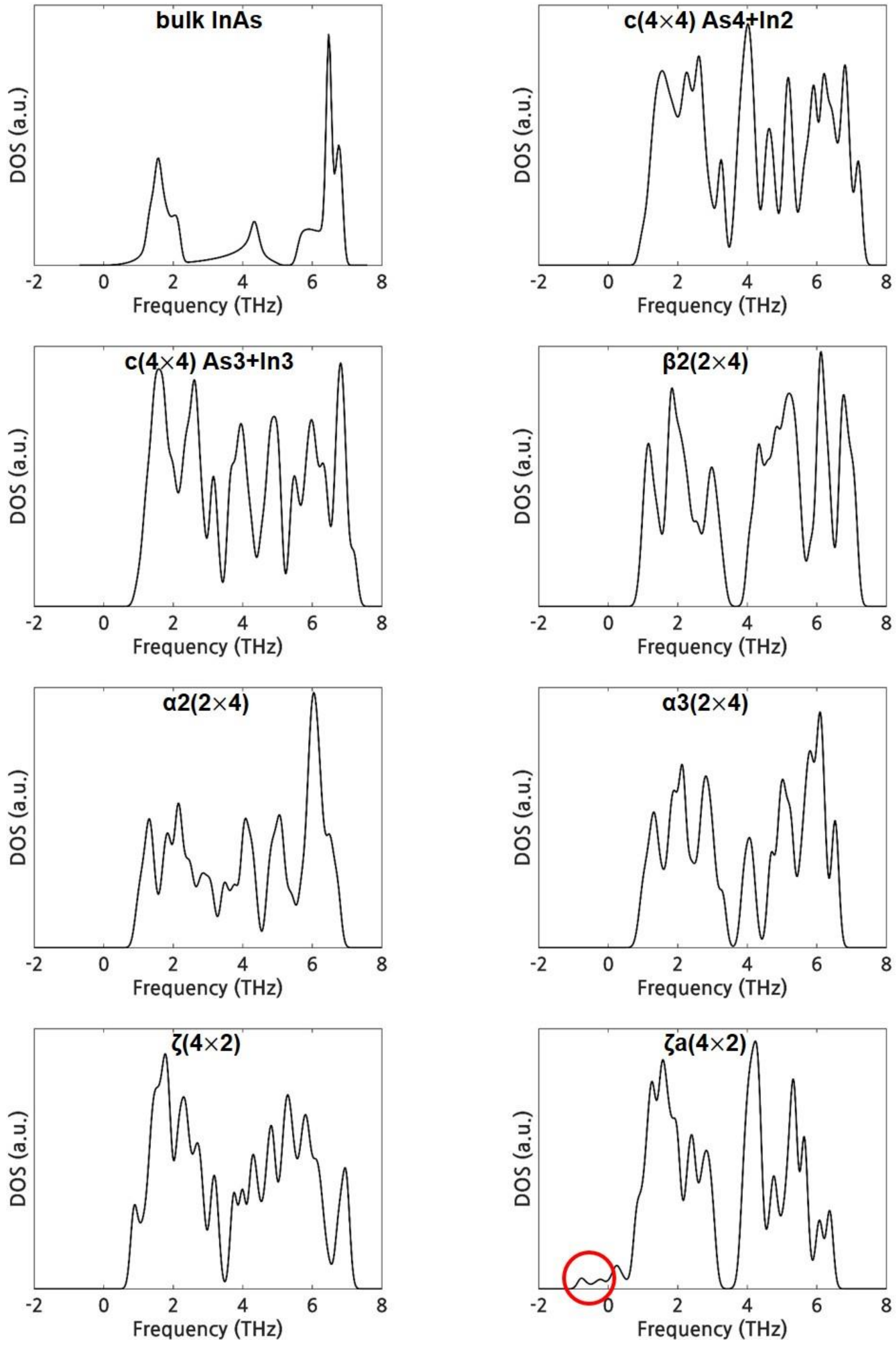
bulk InAs
c(4×4) As4+In2
c(4×4) As3+In3
β2(2×4)
α2(2×4)
α3(2×4)
ζ(4×2)
ζa(4×2)
DOS (a.u.)
Frequency (THz)
-2
0
2
4
6
8

Figure S2. Calculated surface phonon DOSs of various InAs (001) reconstructions. Only the reconstructions with low surface energy are shown. The surface phonon DOSs of the other reconstructions were also calculated in the same way but are not shown. The phonon DOS of the bulk InAs is also shown for comparison.

3. Calculation of the surface energy by the consideration of surface phonons

$$\gamma^{0\mathrm{K}} = \frac{\left[E_{tot}^{\mathrm{II}} - N_{In}^{\mathrm{II}}\mu_{InAs\left(bulk^{0K}\right)} - \left(N_{As}^{\mathrm{II}} - N_{In}^{\mathrm{II}}\right)\mu_{As(InAs)}\right]}{A} - (\gamma_H + \alpha) \qquad (7)$$

From the equation (7), the surface energy at non-0K by the consideration of vibration can be calculated using the following equation:

$$\gamma^{T} = \frac{\left[\left\{E_{tot}^{\mathrm{II}} + F_{vib}^{\mathrm{II}}\right\} - N_{In}^{\mathrm{II}}\left\{\mu_{InAs\left(bulk^{0K}\right)} + F_{vib,InAs(bulk)}\right\} - \left(N_{As}^{\mathrm{II}} - N_{In}^{\mathrm{II}}\right)\left\{\mu_{As(InAs)} + F_{vib,As(bulk)}\right\}\right]}{A} - (\gamma_H + \alpha) \qquad (\mathrm{S.}\,2)$$

where F_{vib}^{II} is the vibrational free energy of the total slab, $F_{vib,InAs(bulk)}$ and $F_{vib,As(bulk)}$ are the vibrational free energy of the bulk InAs and bulk As, respectively.

However, the calculation of phonons of the total slab is time-consuming. Under the assumption that the vibration of the atoms below the three topmost layers are the same as that of the atoms in the bulk state, the γ^{T} for each reconstruction could be calculated using the phonons of only the three topmost layers:

$$\gamma^T = \frac{\{E_{tot}^{\text{II}}+F_{vib,surf}^{\text{II}}+F_{vib,bottom}^{\text{II}}\}}{A} - \frac{\left(N_{In,surf}^{\text{II}}+N_{In,bottom}^{\text{II}}\right)\left\{\mu_{InAs\left(bulk^{0K}\right)}+F_{vib,InAs(bulk)}\right\}}{A} - \frac{\left\{\left(N_{As,surf}^{\text{II}}+N_{As,bottom}^{\text{II}}\right)-\left(N_{In,surf}^{\text{II}}+N_{In,bottom}^{\text{II}}\right)\right\}\{\mu_{As(InAs)}+F_{vib,As(bulk)}\}}{A} - (\gamma_H + \alpha) \quad \text{(S. 3)}$$

where $F_{vib,surf}^{\text{II}}$ is the vibrational free energy of the three topmost layers and $F_{vib,bottom}^{\text{II}}$ is the vibrational free energy of atoms below the three topmost layers ($F_{vib}^{\text{II}} = F_{vib,surf}^{\text{II}} + F_{vib,bottom}^{\text{II}}$), respectively. $N_{In,surf}^{\text{II}}$, $N_{As,surf}^{\text{II}}$ are the numbers of In and As atoms on the three topmost layers and $N_{In,bottom}^{\text{II}}$, $N_{As,bottom}^{\text{II}}$ are the numbers of In and As atoms below the three topmost layers ($N_{In}^{\text{II}} = N_{In,surf}^{\text{II}} + N_{In,bottom}^{\text{II}}$ and $N_{As}^{\text{II}} = N_{As,surf}^{\text{II}} + N_{As,bottom}^{\text{II}}$). The assumption that the vibration of the atoms below the three topmost layers are the same as that of the atoms in the bulk state means:

$$F_{vib,bottom}^{\text{II}} - N_{In,bottom}^{\text{II}} F_{vib,InAs(bulk)} - \left(N_{As,bottom}^{\text{II}} - N_{In,bottom}^{\text{II}}\right) F_{vib,As(bulk)} = 0 \quad \text{(S. 4)}$$

then, the remaining terms in equation (S. 3) is given by:

$$\gamma^{\text{T}} = \gamma^{0\text{K}} + \frac{\left[F_{vib,surf}^{\text{II}} - N_{In,surf}^{\text{II}} F_{vib,InAs(bulk)} - \left(N_{As,surf}^{\text{II}} - N_{In,surf}^{\text{II}}\right) F_{vib,As(bulk)}\right]}{A} \quad (8)$$

4. Effects of configurational entropy and vibrational entropy

Figure S3 shows the fraction of the dominant InAs (001) reconstructions at a fixed P of 4×10^{-9} atm as a function of T. Figure S3 (a) is result including only ‘As3+In3’ and ‘As4+In2’ without considering the surface phonon, while Fig. S3 (b) is that including all the c(4×4) heterodimers without considering the surface phonon. This two figures reveal that all the c(4×4) heterodimers boost the fraction of c(4×4) at the low T region. On the other hand, by considering

the surface phonon as in Fig. S3 (c) and (d), the ζa(4×2) was eliminated due to its instability. By comparing with Fig. 6 (b) (including only c(4×4) homodimers), Fig. S3 (c) including the dominant c(4×4) heterodimers of ‘As3+In3’ and ‘As4+In2’ show the noticeable fraction of the c(4×4) at the low T region. Figure S3 (d) (which is the same as Fig. 6 (c)) show the higher fraction of the c(4×4) by including all the c(4×4) heterodimers.

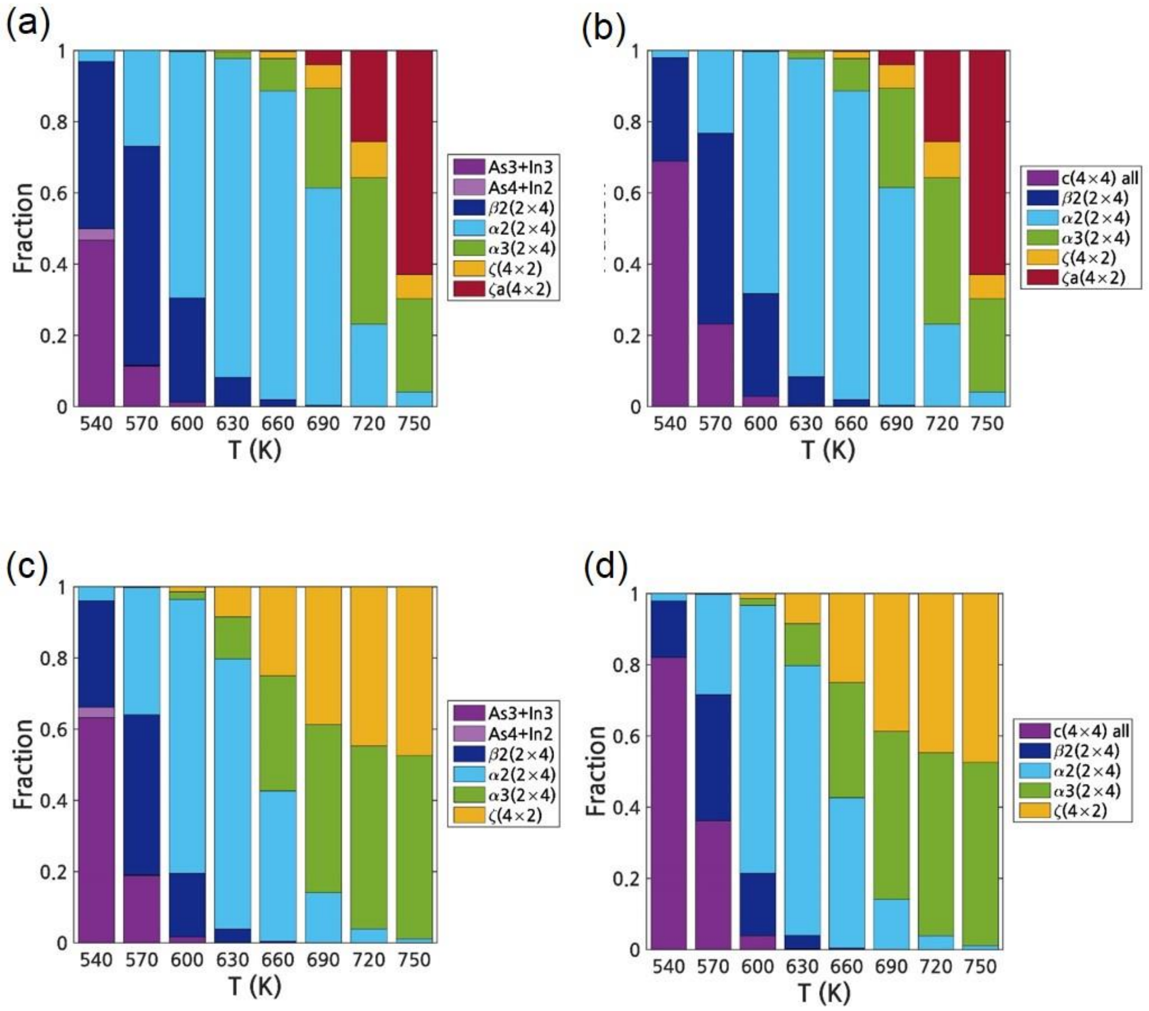

Fig. S3. Fraction of the dominant InAs (001) reconstructions at a fixed P of 4×10^{-9} atm as a function of T. Before considering the surface phonon (a) including only ‘As3+In3’ and ‘As4+In2’, (b) including all the c(4×4) heterodimers. After considering the surface phonon, (c) including only ‘As3+In3’ and ‘As4+In2’and (d) including all the c(4×4) heterodimers.
